# Supplementary material for: Mechano-boosting nanomedicine antitumour efficacy by blocking the reticuloendothelial system with stiff nanogels
Source: Nat Commun. 2023 Mar 15;14:1437. doi: 10.1038/s41467-023-37150-3 (PMC10015032; doi:10.1038/s41467-023-37150-3)
Supplement: Supplementary file 1 — Supplementary Information [file 41467_2023_37150_MOESM1_ESM.pdf]

Supplementary Information for

Mechano-boosting nanomedicine antitumour efficacy by blocking the reticuloendothelial system with stiff nanogels

Zheng Li[1], Yabo Zhu[1], Haowen Zeng[1], Chong Wang[1], Chen Xu[1], Qiang Wang[1], Huimin Wang[1], Shiyou Li[1], Jitang Chen[1], Chen Xiao[1], Xiangliang Yang[1, 2, 3, 4] and Zifu Li[1, 2, 3, 5, *]

[1] National Engineering Research Center for Nanomedicine, College of Life Science and Technology, Huazhong University of Science and Technology, Wuhan, 430074, P. R. China

[2] Key Laboratory of Molecular Biophysics of Ministry of Education, College of Life Science and Technology, Huazhong University of Science and Technology, Wuhan, 430074, P. R. China

[3] Hubei Key Laboratory of Bioinorganic Chemistry and Materia Medical, Huazhong University of Science and Technology, Wuhan, 430074, P. R. China

[4] GBA Research Innovation Institute for Nanotechnology, Guangdong, 510530, P. R. China

[5] Hubei Engineering Research Center for Biomaterials and Medical Protective Materials, Huazhong University of Science and Technology, Wuhan, 430074, P. R. China

* Address correspondence to:

Zifu Li, Ph.D., Professor

Huazhong University of Science and Technology

1037 Luoyu Road, Wuhan, 430074, P. R. China

E-mail: zifuli@hust.edu.cn

Supplementary Tables

Supplementary Table 1. Relative fluorescent intensity of Rhodamine-labeled nanogels with distinctive stiffness at same mass.

	2%NGs	5%NGs	10%NGs	15%NGs
Relative fluorescent intensity	1	0.97	1.93	2.05

Supplementary Table 2. Pharmacokinetic parameters of ICG-loaded 2%NGs with or without RES-blockade. $t_{1/2}$, half-life time; AUC, area under the curve; Vd, apparent volume of distribution; CL, plasma clearance.

	$t_{1/2}$ (h)	AUC ($mg \cdot L^{-1} \cdot h^{-1}$)	Vd ($L \cdot kg^{-1}$)	CL ($L \cdot kg^{-1} \cdot h^{-1}$)
Control	0.70 ± 0.14	8.90 ± 2.15	0.63 ± 0.03	0.65 ± 0.14
2%-blockade	0.87 ± 0.41	9.84 ± 4.58	0.70 ± 0.03	0.63 ± 0.25
15%-blockade	2.29 ± 0.36	41.49 ± 8.71	0.45 ± 0.04	0.14 ± 0.03

Supplementary Table 3. Mass and molar ratio to NIPMAM of components in nanogels with different stiffness.

Nanogels	NIPMAM	BAC	MAA	SDS	KPS
2%NGs	550 mg	22.5 mg (2%)	18.65 μL (5%)	35 mg	10 mg
5%NGs		56.3 mg (5%)		30 mg	
10%NGs		112.6 mg (10%)		25 mg	
15%NGs		168.9 mg (15%)		20 mg	

Supplementary Table 4. Primers for qPCR.

Gene	Forward primer	Reverse primer
Clta	GAGAGTGAGATTGCGGGCAT	GTCAACAGCATCCGGACCTC
Cltc	GCCAGATGTCGTCCTGGAAA	GCATCTAATTTATCCACCTTGGTCA
Ap2a1	TAATGCCCTCAAGACGGAGC	CGGATGGCATAGTCAGCAGT
Ap2a2	CCAGTGCTGCGTCCACA	TCTGAGAACACGTCCACCAG
Ap2b1	GATGCCCGAGCAGCTATGAT	TTCACTATGGCGGTGAGCAG

Ap2m1	TTCAAGCCCTCACTTCTGGC	TGCCATGCGCTTGATCTTCC
Ap2s1	CACCAACTTTGTGGAGTTCCG	GTTGTGGATGGCCTCGAGAT
GAPDH	TGACAACTTTGGCATTGTGGAA	GTGGATGCAGGGATGATGTTCT

Supplementary Figures

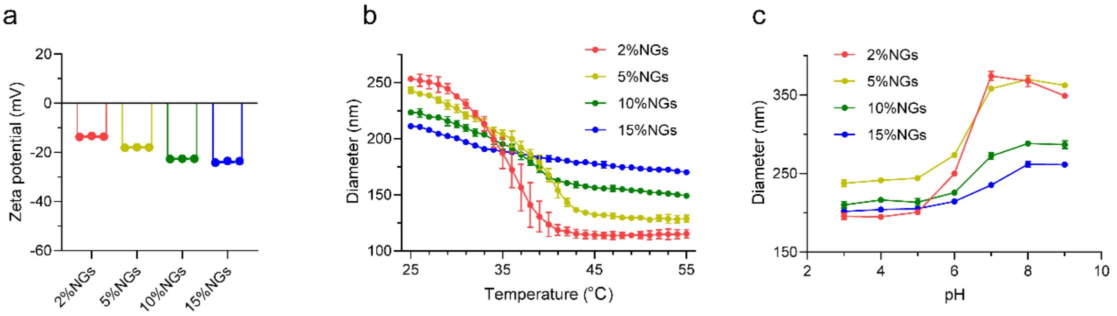

Supplementary Figure 1. Characterization of nanogels with distinctive stiffness. a Zeta-potential of nanogels with different stiffness in H_2O. Data are presented as mean values ± SD (n = 3 independent replicates). **b** Temperature-responsiveness and **c** pH-responsiveness of nanogels with different stiffness in H_2O. Data are presented as mean values ± SD (n = 3 independent replicates). Source data are provided as a Source Data file.

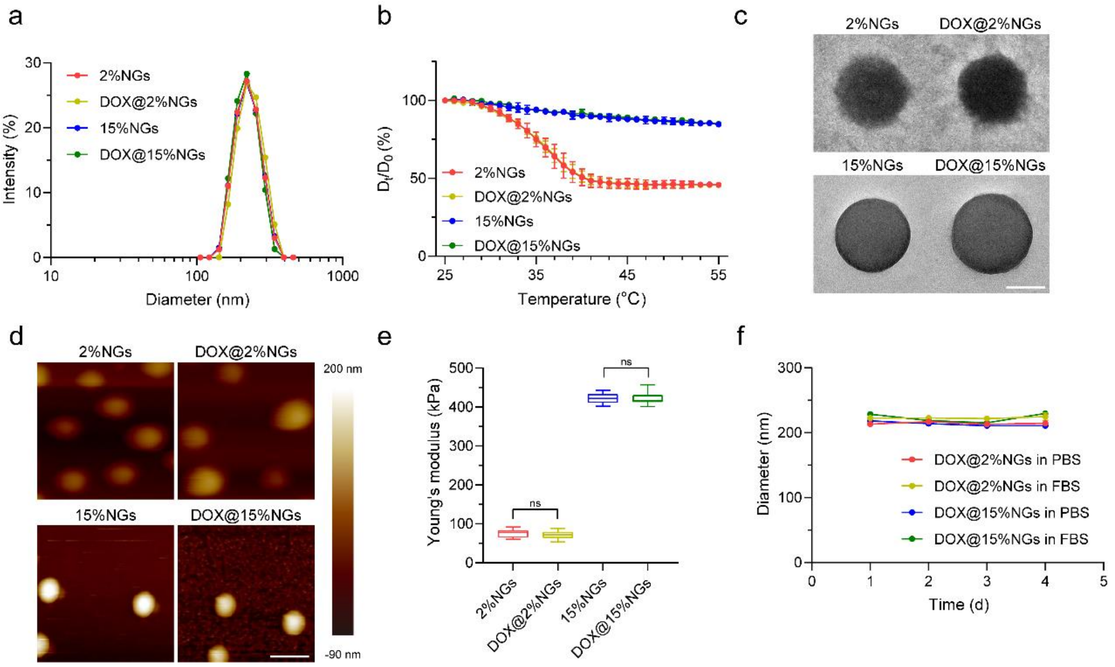

Supplementary Figure 2. Characterization of nanogels with or without loading DOX. a Size distribution of nanogels with different stiffness with or without loading DOX. **b** Temperature-responsiveness of nanogels with different stiffness with or without loading DOX in H_2O. Data are presented as mean values ± SD (n = 3 independent replicates). **c** TEM images of nanogels with different stiffness with or without loading DOX. Scale bar = 100 nm. **d** AFM images of nanogels with different stiffness with or without loading DOX.

Scale bar = 200 nm. **e** Young's modulus of nanogels with different stiffness with or without loading DOX. Box plots indicate median (middle line), 25th, 75th percentile (box) and minimum and maximum (whiskers) (*n* = 15 independent replicates). **f** Stability of DOX@2%NGs and DOX@15%NGs in PBS or FBS. Data are presented as mean values ± SD (*n* = 3 independent replicates). Statistical significance was calculated by unpaired two-sided Student's t-test. Source data are provided as a Source Data file.

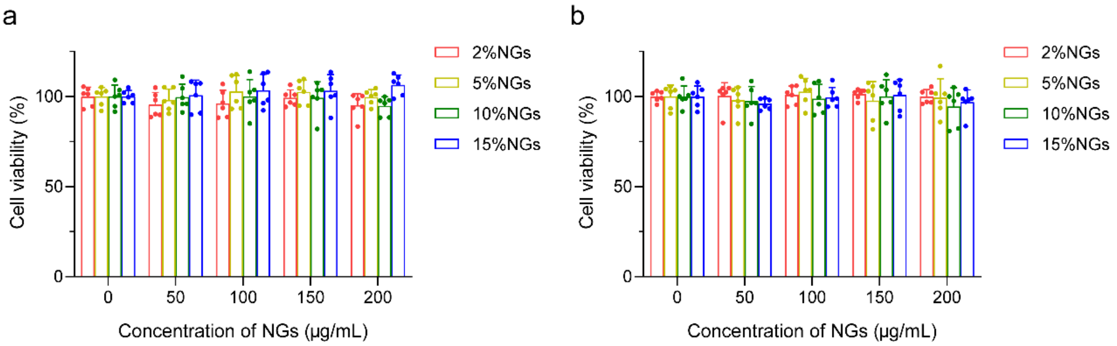

Supplementary Figure 3. Cytotoxicity of nanogels with different stiffness. Cytotoxicity of nanogels with different stiffness for **a** 48 h and **b** 72 h. Data are presented as mean values ± SD (*n* = 6 biological independent replicates). Source data are provided as a Source Data file.

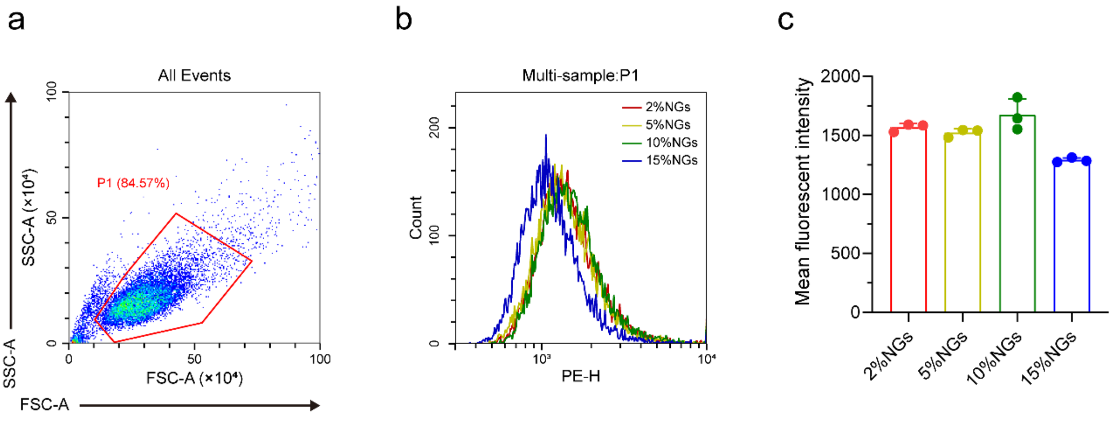

Supplementary Figure 4. Cellular uptake efficiency of nanogels with different stiffness by 4T1 cells. a Gating strategy for cellular uptake. **b** Flow cytometry graphs of 4T1 cells after internalization of Rhodamine-labeled nanogels with different stiffness. **c** Mean fluorescent intensity of 4T1 cells after internalization of Rhodamine-labeled nanogels with different stiffness. Data are presented as mean values ± SD (*n* = 3 biological

independent replicates). Source data are provided as a Source Data file.

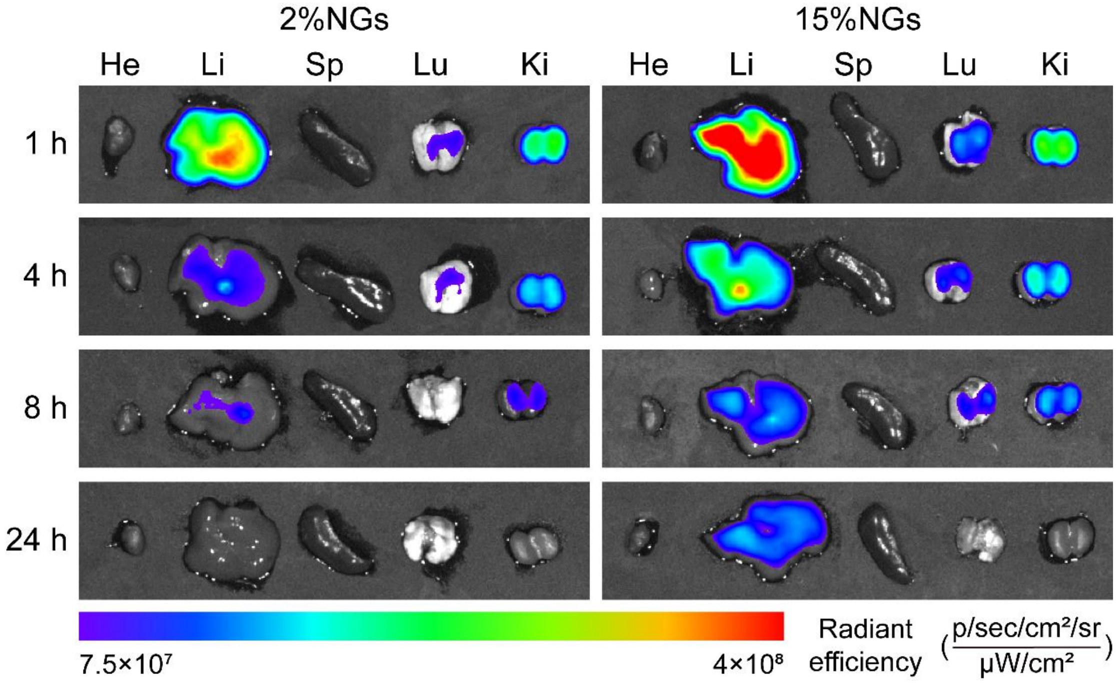

Supplementary Figure 5. Ex vivo fluorescent images of ICG-loaded nanogels with different stiffness in major organs. He, heart; Li, liver; Sp, spleen; Lu, lung; Ki, kidney.

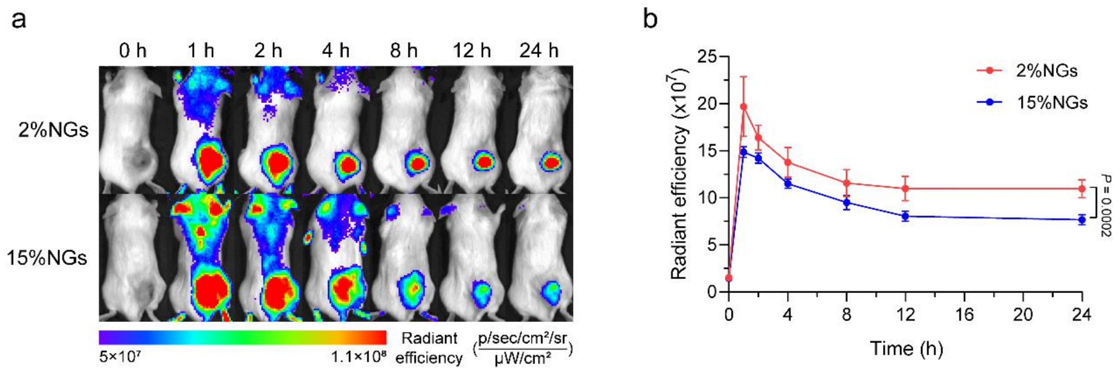

Supplementary Figure 6. Tumour accumulation of ICG-loaded nanogels with different stiffness. a In vivo fluorescent images of tumour accumulation of ICG-loaded nanogels with different stiffness. **b** Semi-quantification of the quantity of ICG-loaded nanogels with different stiffness in tumour. Data are presented as mean values ± SD (n = 5 biological independent replicates). Statistical significance was calculated by unpaired two-sided Student's t-test. Source data are provided as a Source Data file.

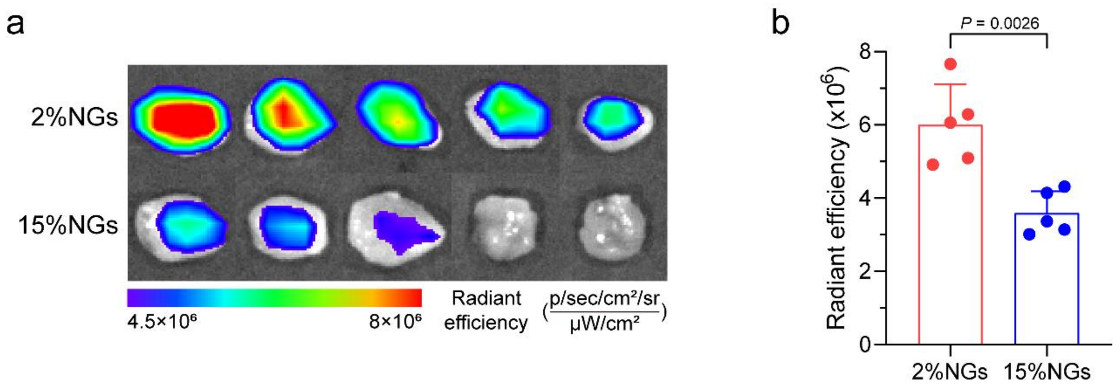

Supplementary Figure 7. Tumour accumulation of Rhodamine-labeled nanogels with different stiffness. a Ex vivo fluorescent images of tumour accumulation of Rhodamine-labeled nanogels with different stiffness. **b** Semi-quantification of the quantity of Rhodamine-labeled nanogels with different stiffness in tumour. Data are presented as mean values ± SD (*n* = 5 biological independent replicates). Statistical significance was calculated by unpaired two-sided Student's t-test. Source data are provided as a Source Data file.

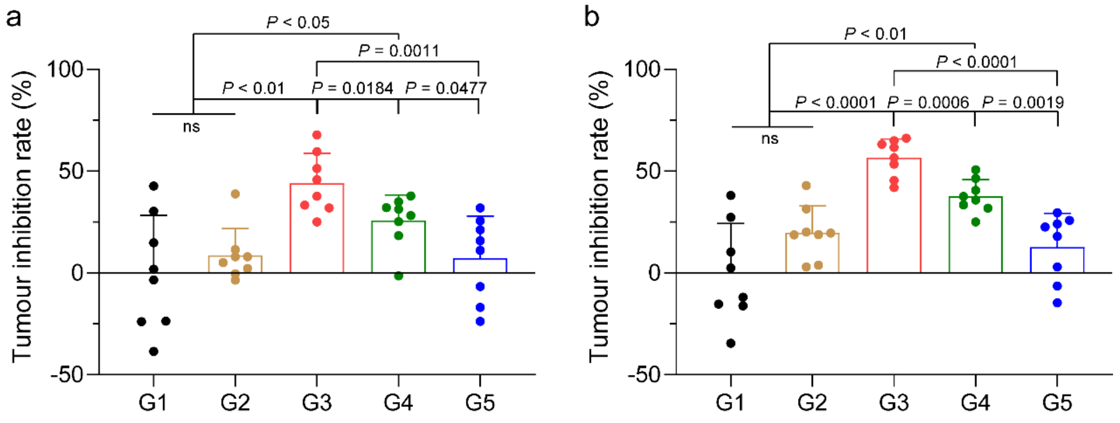

Supplementary Figure 8. Tumour inhibition rate of DOX-loaded nanogels with different stiffness. a Volume-based and **b** weight-based tumour inhibition rate. Data are presented as mean values ± SD (*n* = 8 biological independent replicates). Statistical significance was calculated by unpaired two-sided Student's t-test. G1, Control; G2, free DOX; G3, DOX@2%NGs; G4, DOX@10%NGs; G5, DOX@15%NGs. Source data are provided as a Source Data file.

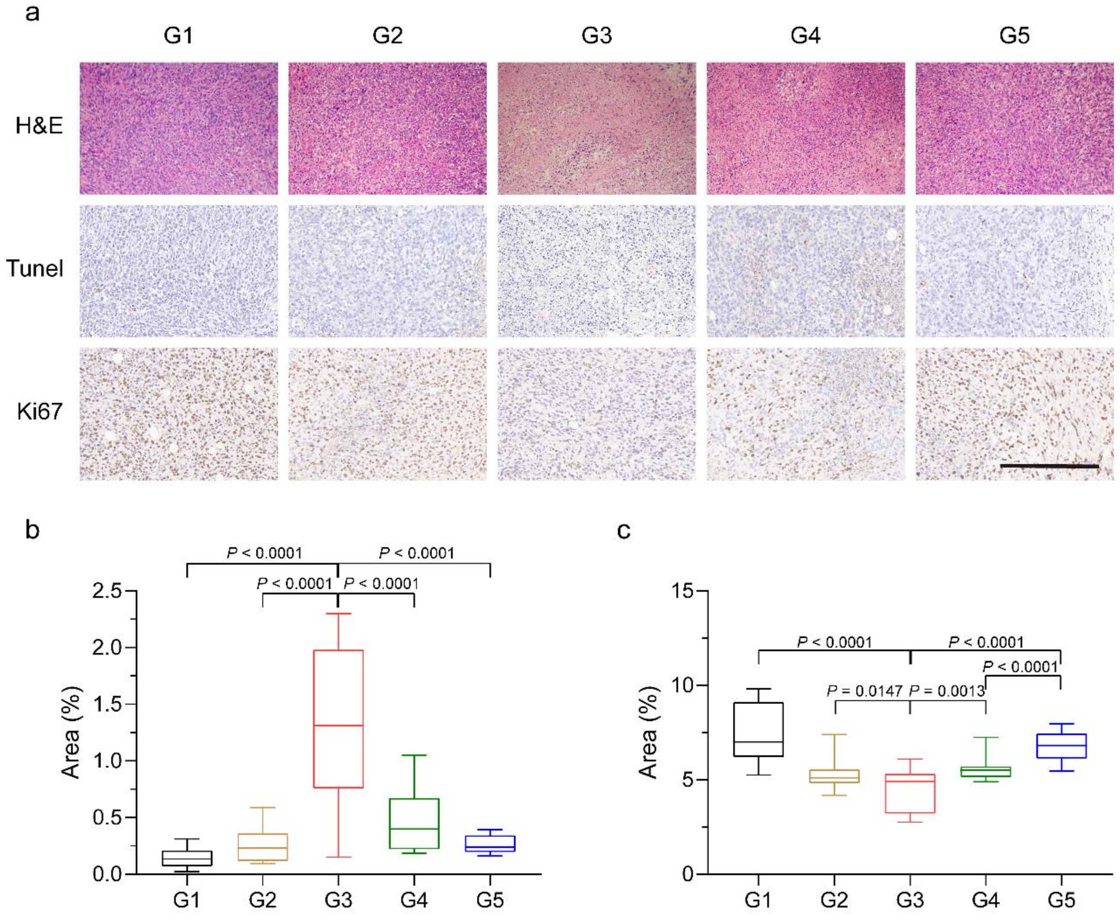

Supplementary Figure 9. Apoptosis and proliferation of tumour cells after treatment by DOX-loaded nanogels with different stiffness. a H&E, Tunel and Ki67 staining of tumours. Scale bar = 500 μm. Percentage of the area of **b** apoptotic cells and **c** proliferative cells. Box plots indicate median (middle line), 25th, 75th percentile (box) and minimum and maximum (whiskers) (*n* = 15 independent replicates). Statistical significance was calculated by unpaired two-sided Student's t-test. G1, Control; G2, free DOX; G3, DOX@2%NGs; G4, DOX@10%NGs; G5, DOX@15%NGs. Source data are provided as a Source Data file.

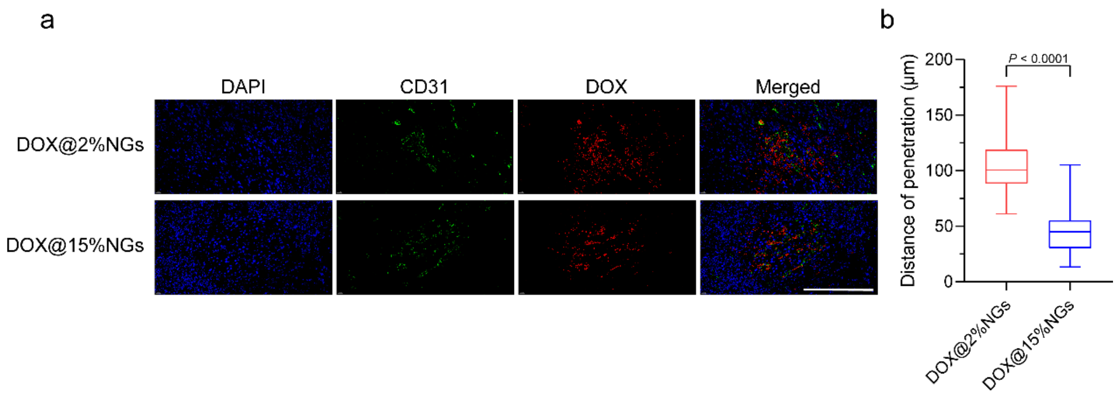

Supplementary Figure 10. Tumour penetration of nanogels with different stiffness. a CD31 staining of tumours. Blue: nuclei of tumour cells. Green: blood vessels. Red: DOX-loaded nanogels. Scale bar = 500 μm. **b** Distance from DOX-loaded nanogels with different stiffness to the nearest blood vessel. Box plots indicate median (middle line), 25th, 75th percentile (box) and minimum and maximum (whiskers) (*n* = 80 independent replicates). Statistical significance was calculated by unpaired two-sided Student's t-test. Source data are provided as a Source Data file.

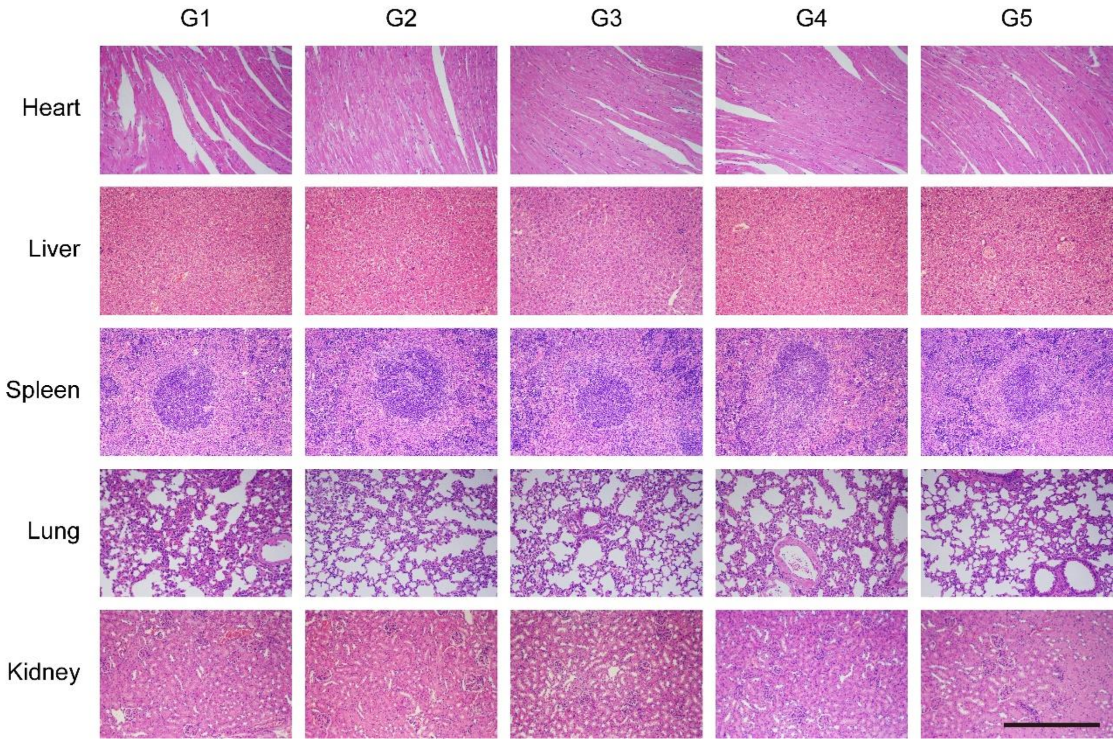

Supplementary Figure 11. Safety of treatment by DOX-loaded nanogels with different stiffness. H&E staining of major organs, including heart, liver, spleen, lung and kidney. Scale bar = 500 μm. G1, Control; G2, free DOX; G3, DOX@2%NGs; G4, DOX@10%NGs;

G5, DOX@15%NGs.

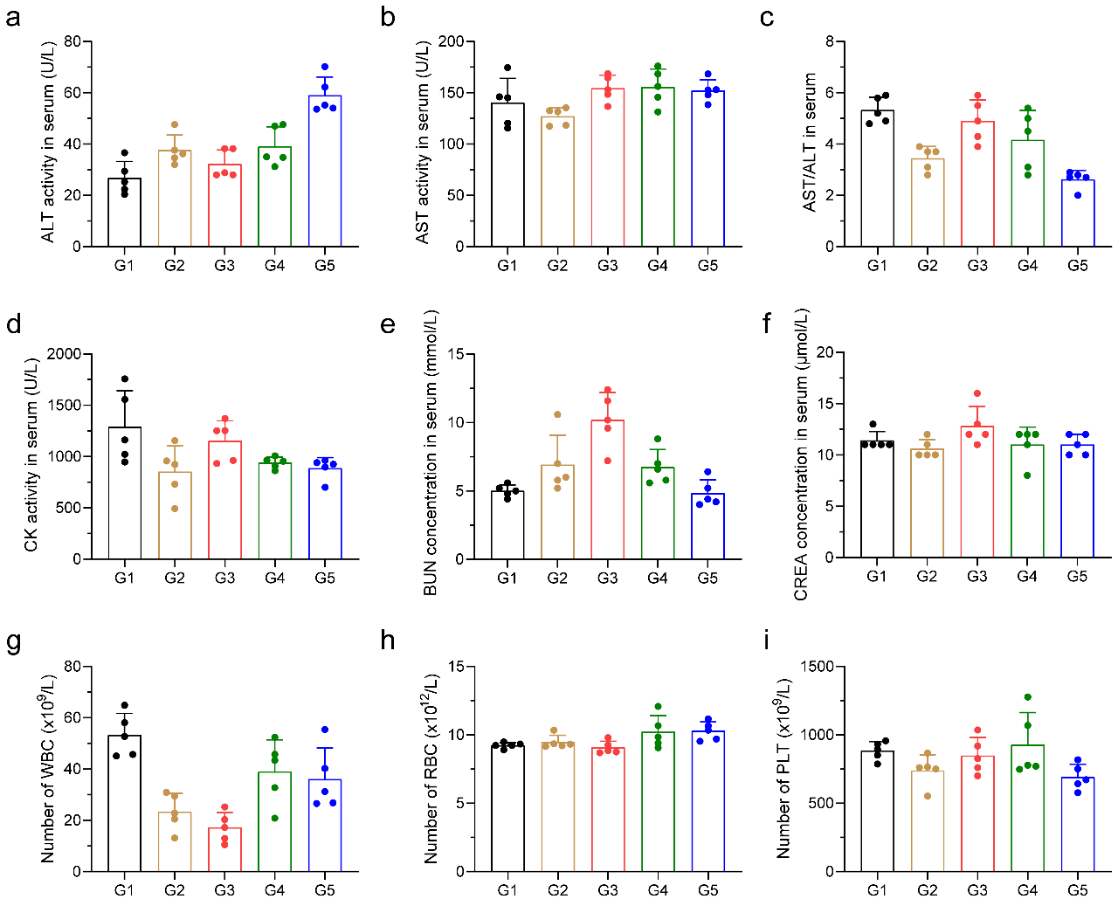

Supplementary Figure 12. Safety of treatment by DOX-loaded nanogels with different stiffness. a-f Blood biochemical analysis and **g-i** blood routine examine after treatment. Data are presented as mean values ± SD (n = 5 biological independent replicates). G1, Control; G2, free DOX; G3, DOX@2%NGs; G4, DOX@10%NGs; G5, DOX@15%NGs. Source data are provided as a Source Data file.

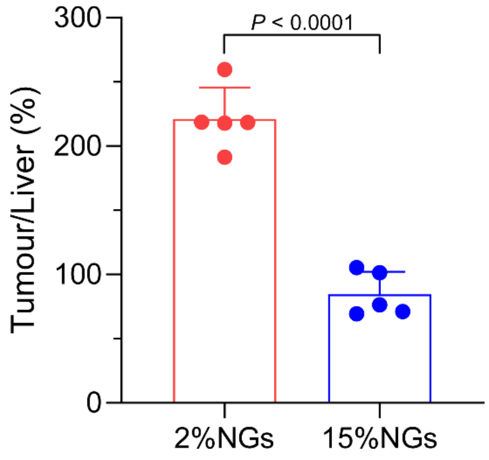

Supplementary Figure 13. Relative accumulation of tumour to liver of ICG-loaded nanogels with different stiffness. Data are presented as mean values ± SD (n = 5 biological independent replicates). Statistical significance was calculated by unpaired two-sided Student's t-test. Source data are provided as a Source Data file.

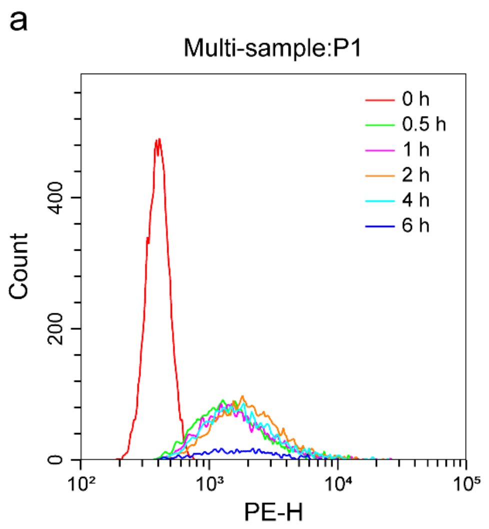

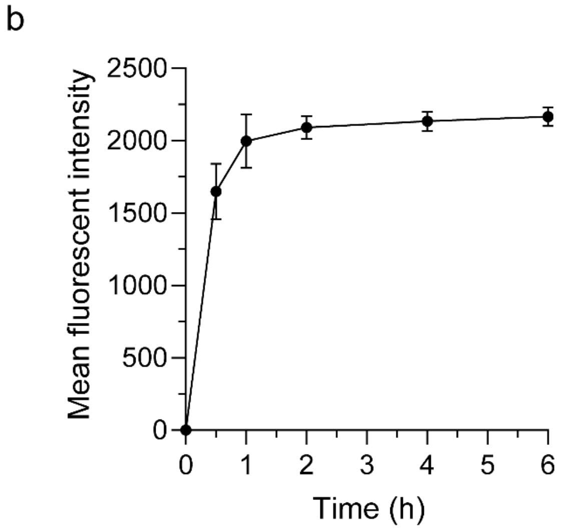

Supplementary Figure 14. Cellular uptake of Rhodamine-labeled nanogels with different stiffness by RAW 264.7 cells. a Flow cytometry graphs of RAW 264.7 cells after internalization of Rhodamine-labeled 15%NGs. **b** Cellular uptake of Rhodamine-labeled 15%NGs by RAW 264.7 cells. Data are presented as mean values ± SD (n = 3 biological independent replicates). Source data are provided as a Source Data file.

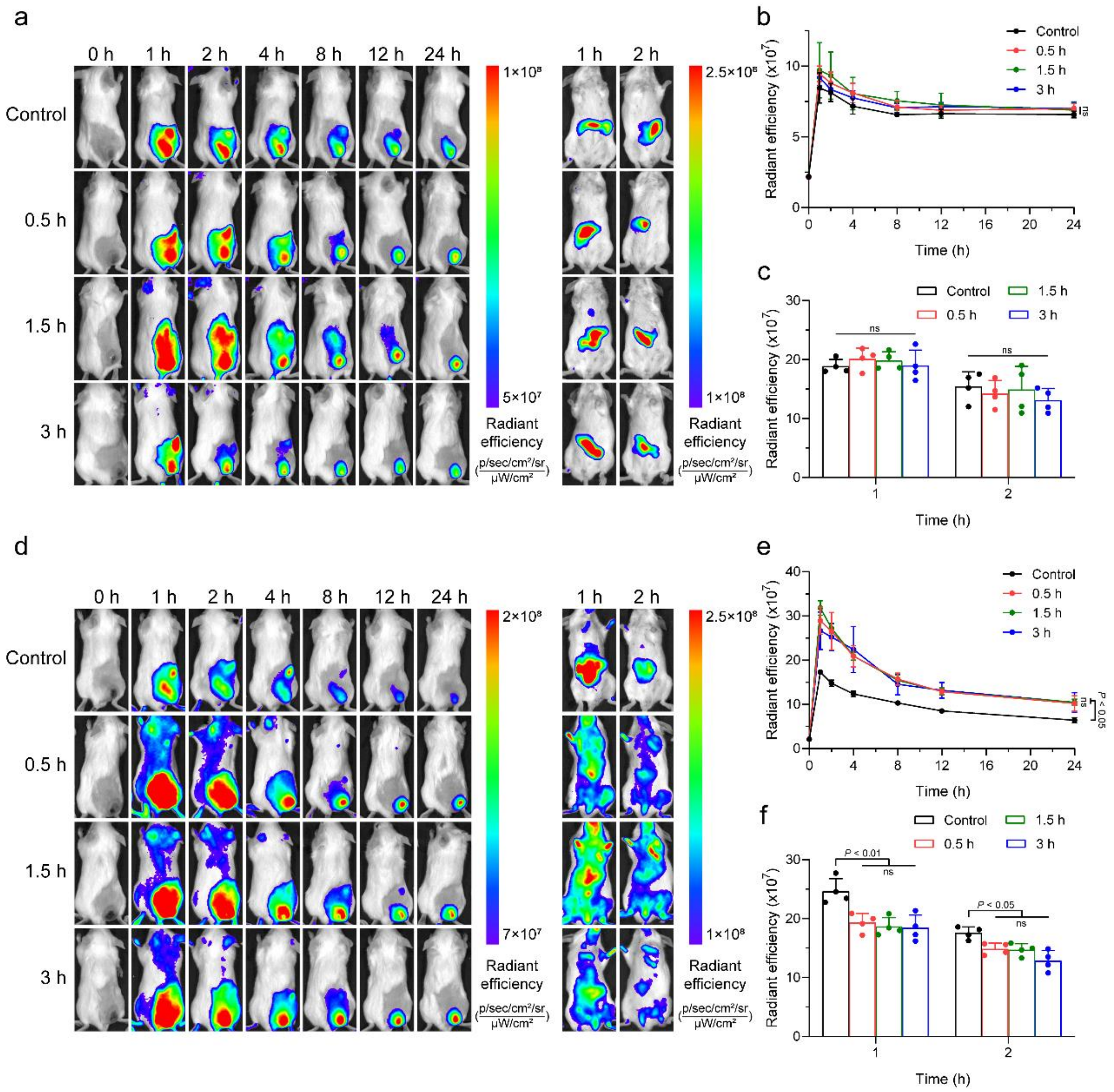

Supplementary Figure 15. Biodistribution of ICG-loaded 2%NGs after RES-blockade with different time interval. a Tumour accumulation and liver accumulation of ICG-loaded 2%NGs after RES-blockade by 2%NGs with different time interval. Semi-quantification of the quantity of ICG-loaded 2%NGs in **b** tumour and **c** liver. Data are presented as mean values ± SD (*n* = 4 biological independent replicates). **d** Tumour accumulation and liver accumulation of ICG-loaded 2%NGs after RES-blockade by 15%NGs with different time interval. Semi-quantification of the quantity of ICG-loaded 2%NGs in **e** tumour and **f** liver. Data are presented as mean values ± SD (*n* = 4 biological independent replicates). Statistical significance was calculated by one-way ANOVA. Source data are provided as a Source Data file.

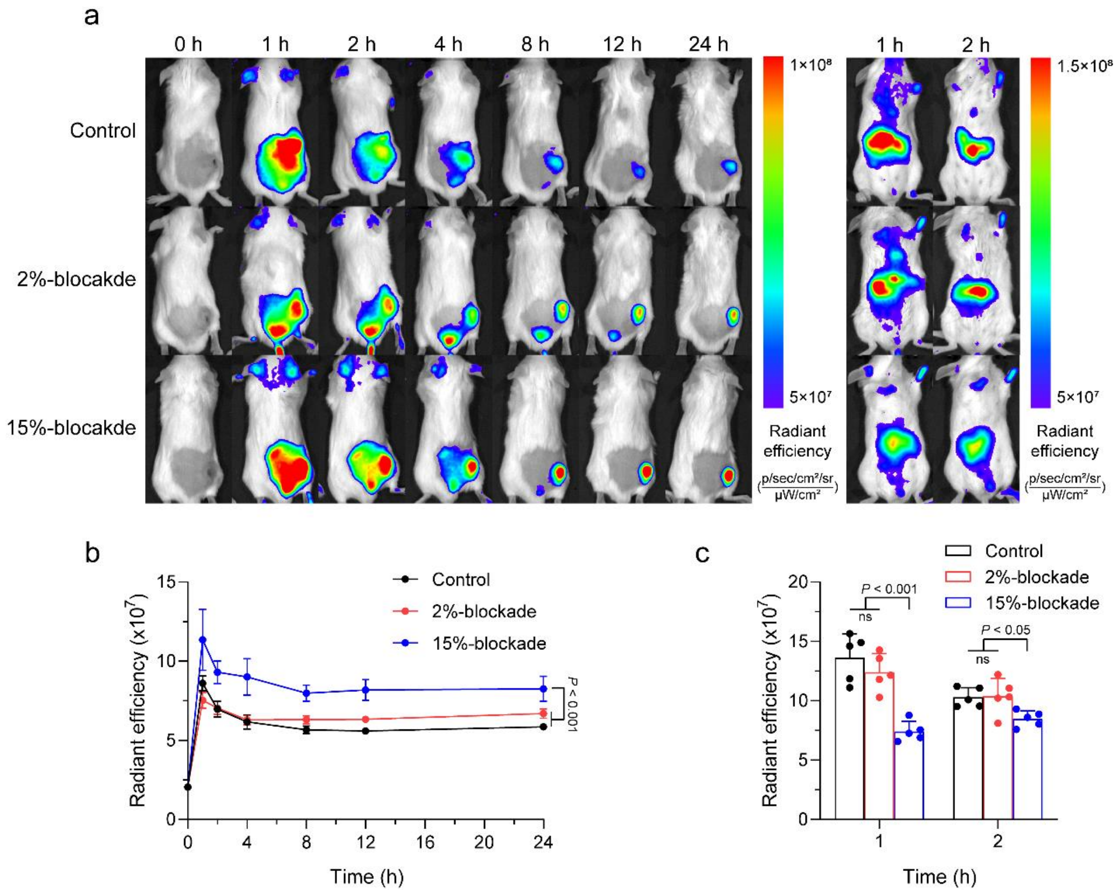

Supplementary Figure 16. Biodistribution of ICG-loaded 2%NGs after RES-blockade by nanogels with different stiffness. a In vivo fluorescent images of tumour accumulation and liver accumulation of ICG-loaded 2%NGs after RES-blockade by nanogels with different stiffness at dosage of 200 mg/kg. Semi-quantification of the quantity of ICG-loaded 2%NGs in **b** tumour and **c** liver. Data are presented as mean values ± SD (*n* = 5 biological independent replicates). Statistical significance was calculated by one-way ANOVA. Source data are provided as a Source Data file.

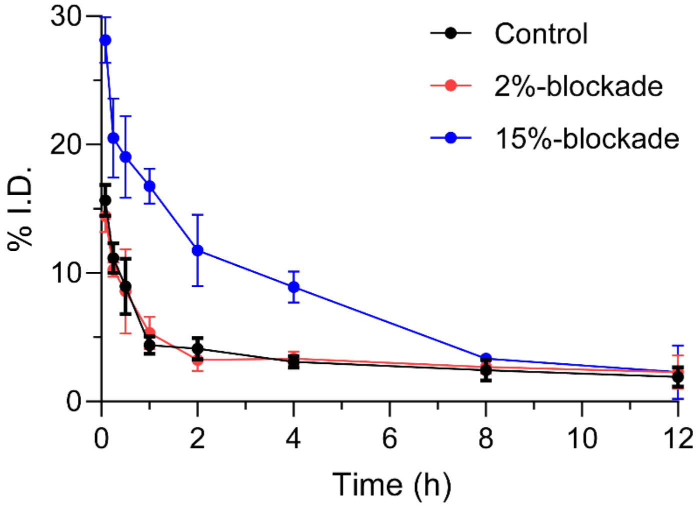

Supplementary Figure 17. Pharmacokinetics of ICG-loaded 2%NGs with or without RES-blockade. Data are presented as mean values ± SD (*n* = 3 biological independent replicates). Source data are provided as a Source Data file.

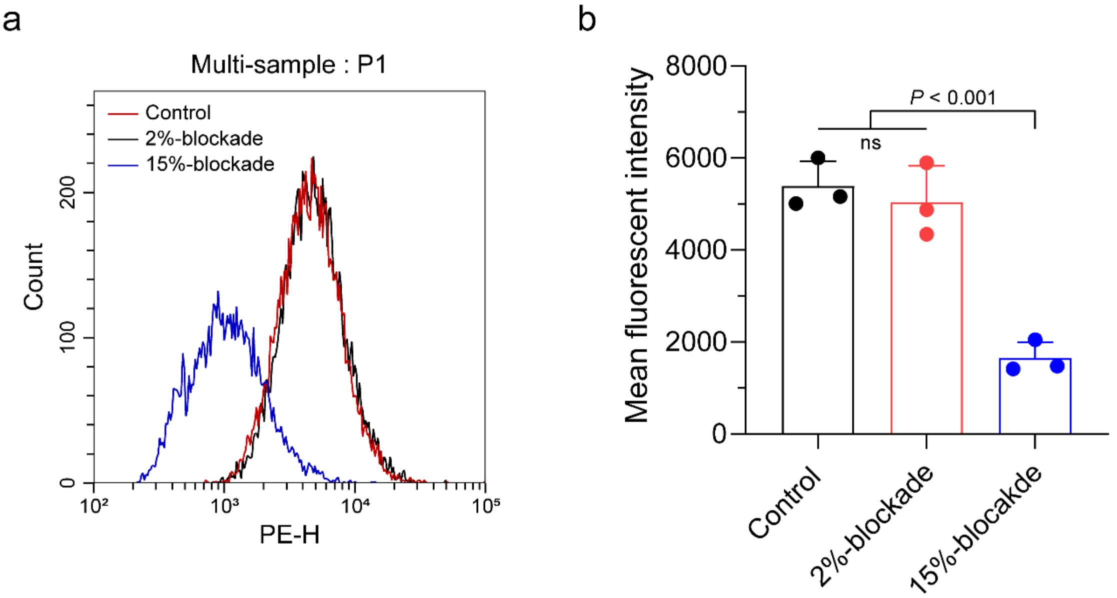

Supplementary Figure 18. Cellular uptake of Rhodamine-labeled 2%NGs after blockade by nanogels with different stiffness. a Flow cytometry graphs of RAW 264.7 cells internalizing Rhodamine-labeled 2%NGs with or without RES-blockade. **b** Mean fluorescent intensity of RAW 264.7 cells internalizing Rhodamine-labeled 2%NGs with or without RES-blockade. Data are presented as mean values ± SD (*n* = 3 biological independent replicates). Statistical significance was calculated by one-way ANOVA. Source data are provided as a Source Data file.

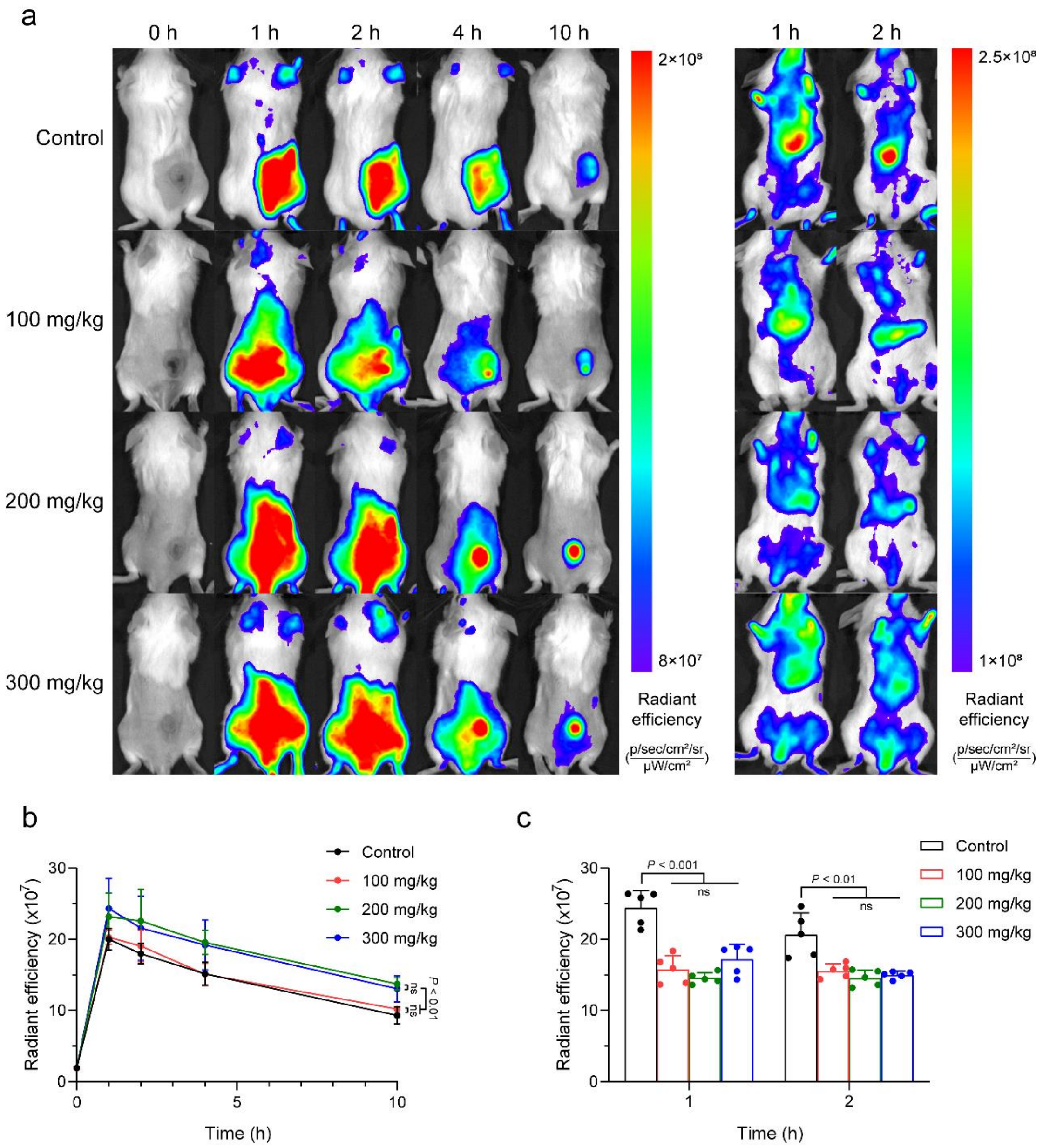

Supplementary Figure 19. Biodistribution of ICG-loaded 2%NGs after RES-blockade by 15%NGs with different dosage. a In vivo fluorescent images of tumour accumulation and liver accumulation of ICG-loaded 2%NGs after RES-blockade by 15%NGs with different dosage. Semi-quantification of the quantity of ICG-loaded 2%NGs in **b** tumour and **c** liver. Data are presented as mean values ± SD (*n* = 5 biological independent replicates). Statistical significance was calculated by one-way ANOVA. Source data are provided as a Source Data file.

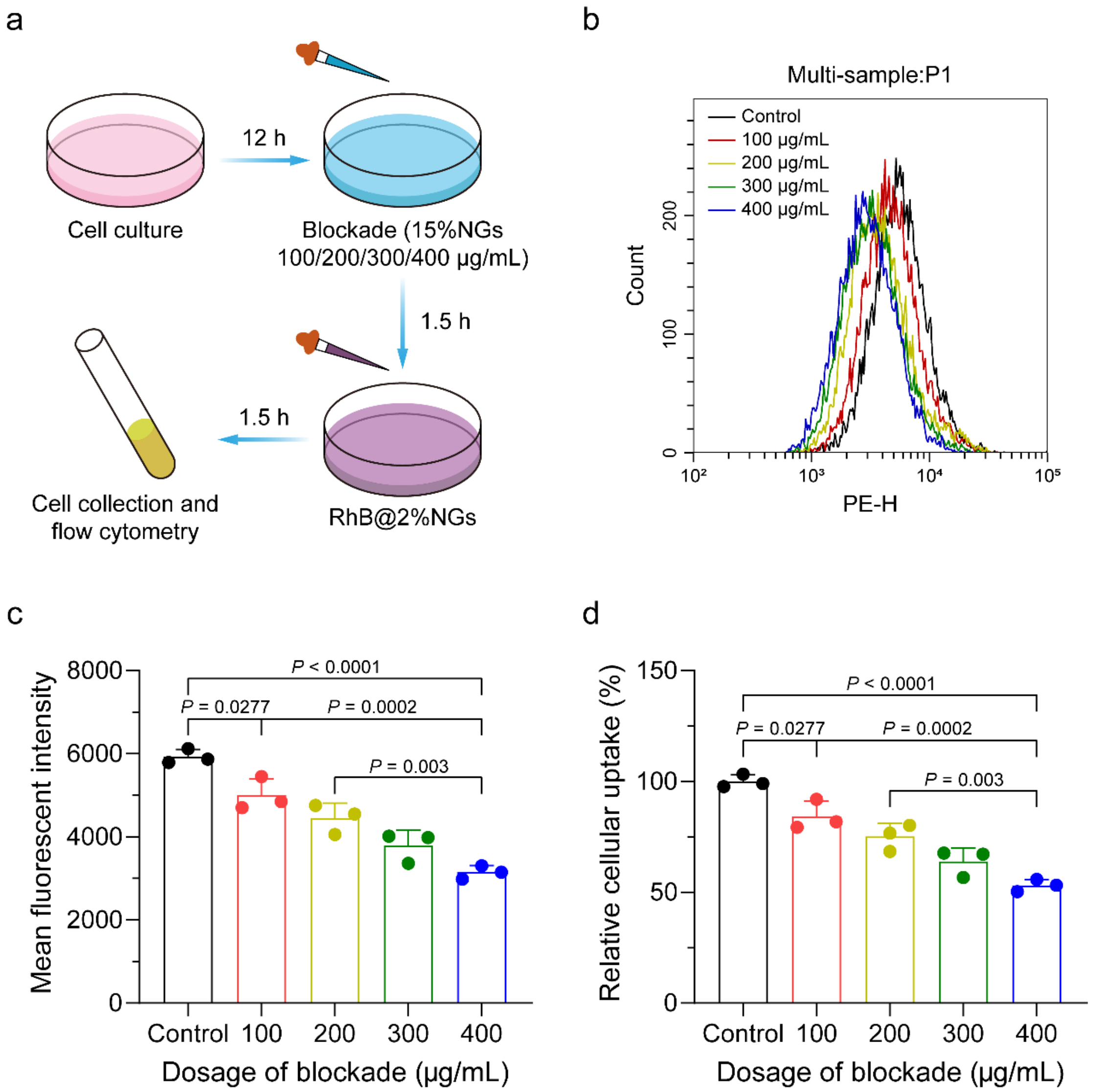

Supplementary Figure 20. Cellular uptake of Rhodamine-labeled 2%NGs after blockade by 15%NGs with different dosage. a Illustration of in vitro dosage-dependent RES-blockade experiment within RAW 264.7 cells. **b** Flow cytometry graphs of RAW 264.7 cells internalizing Rhodamine-labeled 2%NGs after RES-blockade by 15%NGs with different dosage. **c** Mean fluorescent intensity of RAW 264.7 cells internalizing Rhodamine-labeled 2%NGs after RES-blockade by 15%NGs with different dosage. Data are presented as mean values ± SD (*n* = 3 biological independent replicates). **d** Different blockade efficiency of 15%NGs with different dosage in RAW 264.7 cells. Data are presented as mean values ± SD (*n* = 3 biological independent replicates). Statistical significance was calculated by one-way ANOVA. Source data are provided as a Source Data file.

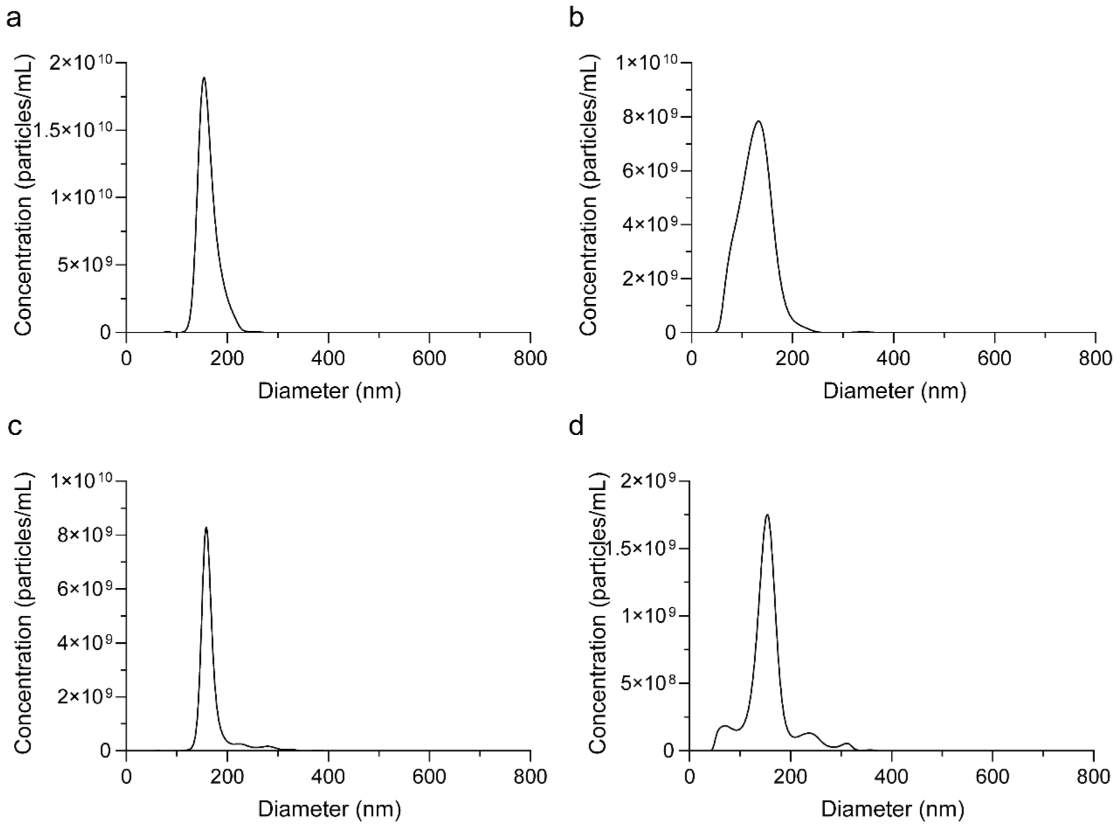

Supplementary Figure 21. NTA analysis of nanogels with different stiffness. Concentration-dependent diameter distribution of nanogels with different stiffness at concentration of 2 mg/mL. **a** 2%NGs, **b** 5%NGs, **c** 10%NGs, **d** 15%NGs. Source data are provided as a Source Data file.

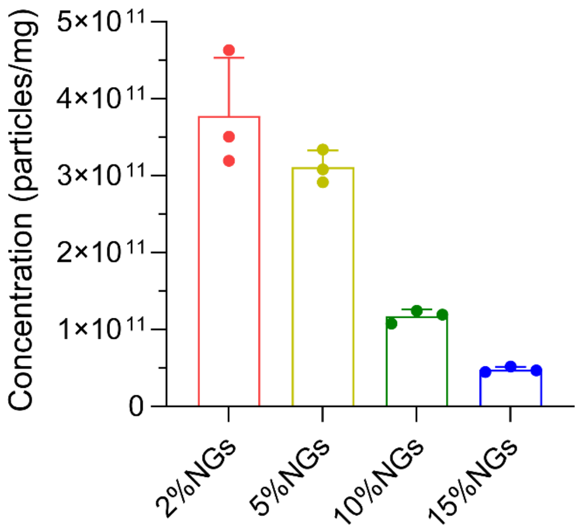

Supplementary Figure 22. Number of nanogels with different stiffness per milligram by NTA. Data are presented as mean values ± SD (n = 3 independent replicates). Source data are provided as a Source Data file.

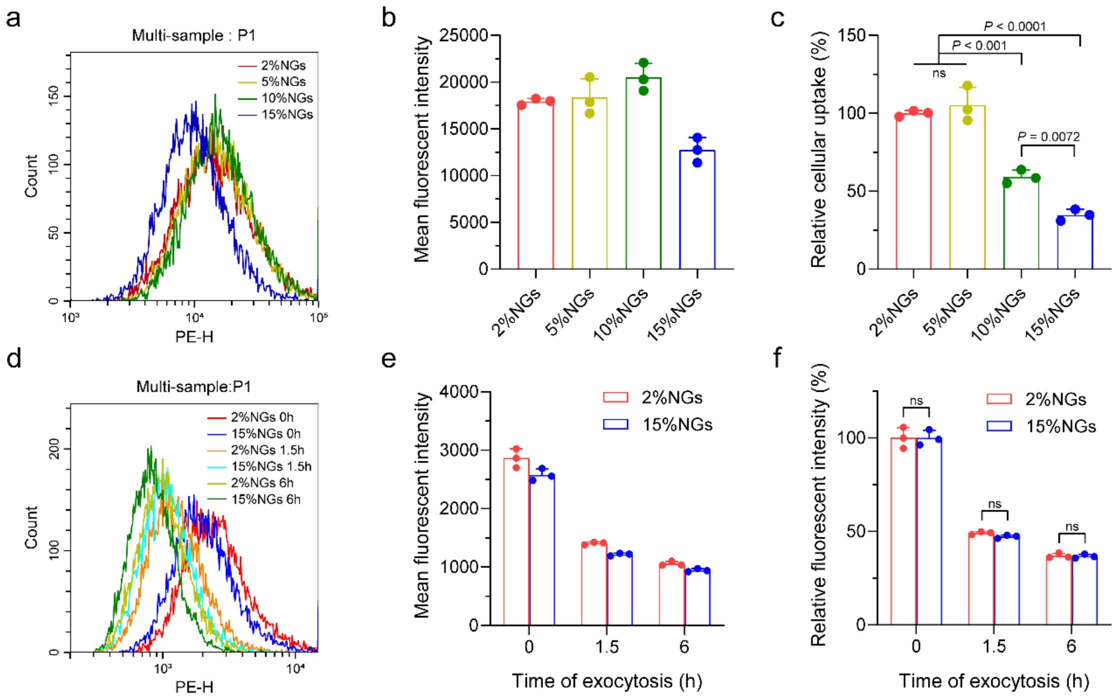

Supplementary Figure 23. Cellular uptake and exocytosis of Rhodamine-labeled nanogels with different stiffness by RAW 264.7 cells. a Flow cytometry graphs of RAW 264.7 cells internalizing Rhodamine-labeled nanogels with different stiffness. **b** Mean fluorescent intensity of RAW 264.7 cells after internalization of Rhodamine-labeled nanogels with different stiffness. Data are presented as mean values ± SD (*n* = 3 biological independent replicates). **c** Relative cellular uptake efficiency of Rhodamine-labeled nanogels with different stiffness in RAW 264.7 cells. Data are presented as mean values ± SD (*n* = 3 biological independent replicates). Statistical significance was calculated by one-way ANOVA. **d** Flow cytometry graphs of RAW 264.7 cells excreting Rhodamine-labeled nanogels with different stiffness. **e** Mean fluorescent intensity of RAW 264.7 cells after excreting Rhodamine-labeled nanogels with different stiffness. Data are presented as mean values ± SD (*n* = 3 biological independent replicates). **f** Relative fluorescent intensity of RAW 264.7 cells after excreting Rhodamine-labeled nanogels with different stiffness. Data are presented as mean values ± SD (*n* = 3 biological independent replicates). Statistical significance was calculated by unpaired two-sided Student's t-test. Source data are provided as a Source Data file.

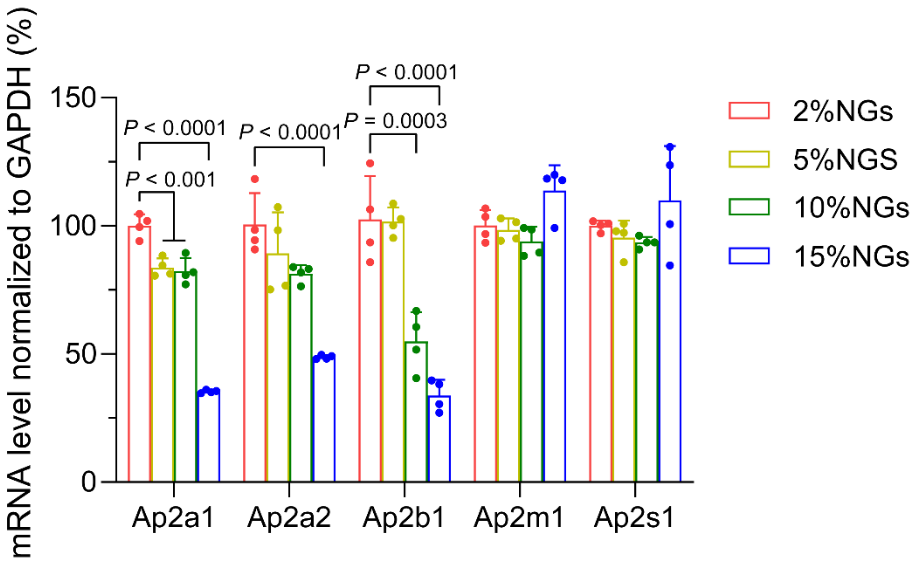

Supplementary Figure 24. AP-2 mRNA as normalized to GAPDH after incubation with nanogels with different stiffness. Data are presented as mean values ± SD (n = 4 biological independent replicates). Statistical significance was calculated by one-way ANOVA. Source data are provided as a Source Data file.

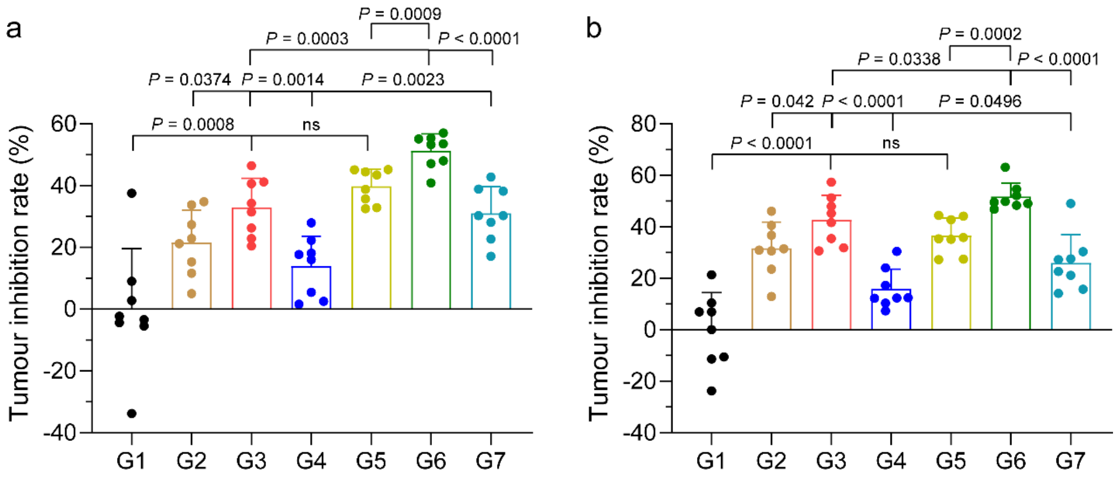

Supplementary Figure 25. Tumour inhibition rate of different combination of RES-blockade and drug delivery strategy. a Volume-based and **b** weight-based tumour inhibition rate. Data are presented as mean values ± SD (n = 8 biological independent replicates). Statistical significance was calculated by unpaired two-sided Student's t-test. G1, Control; G2, Free DOX; G3, DOX@2%NGs; G4, DOX@15%NGs; G5, 2%-blockade + DOX@2%NGs; G6, 15%-blockade + DOX@2%NGs; G7, 15%-blockade + DOX@15%NGs. Source data are provided as a Source Data file.

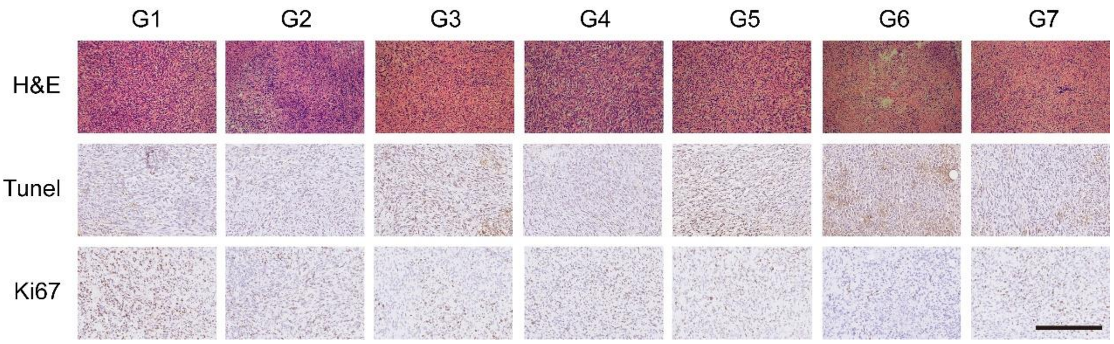

Supplementary Figure 26. Apoptosis and proliferation of tumour cells after treatment by different combination of RES-blockade and drug delivery strategy. H&E, Tunel and Ki67 staining of tumours post various treatments. Scale bar = 500 μm. G1, Control; G2, Free DOX; G3, DOX@2%NGs; G4, DOX@15%NGs; G5, 2%-blockade + DOX@2%NGs; G6, 15%-blockade + DOX@2%NGs; G7, 15%-blockade + DOX@15%NGs.

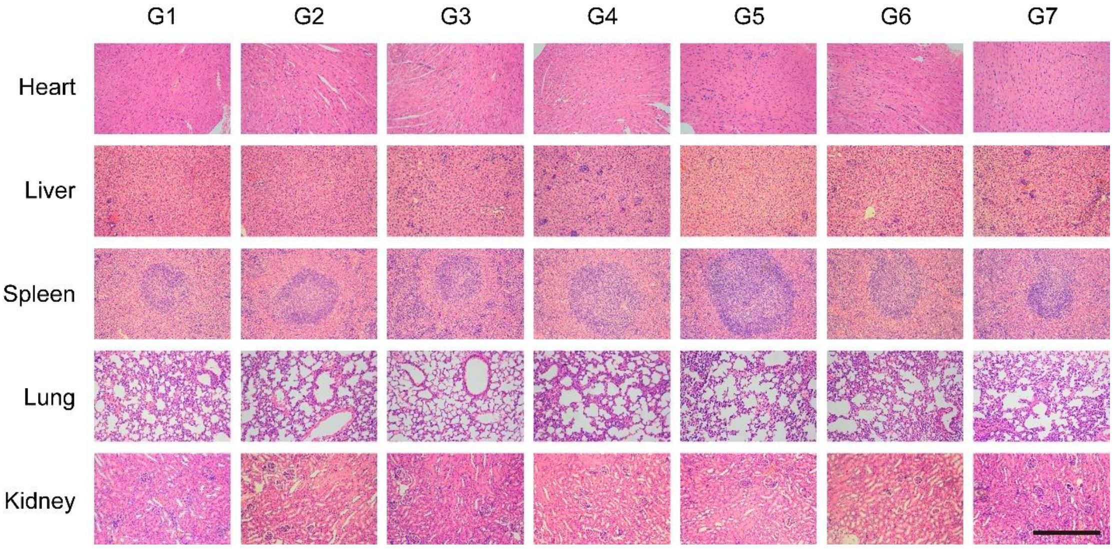

Supplementary Figure 27. Safety of treatment by different combination of RES-blockade and drug delivery strategy. H&E staining of major organs, including heart, liver, spleen, lung and kidney. Scale bar = 500 μm. G1, Control; G2, Free DOX; G3, DOX@2%NGs; G4, DOX@15%NGs; G5, 2%-blockade + DOX@2%NGs; G6, 15%-blockade + DOX@2%NGs; G7, 15%-blockade + DOX@15%NGs.

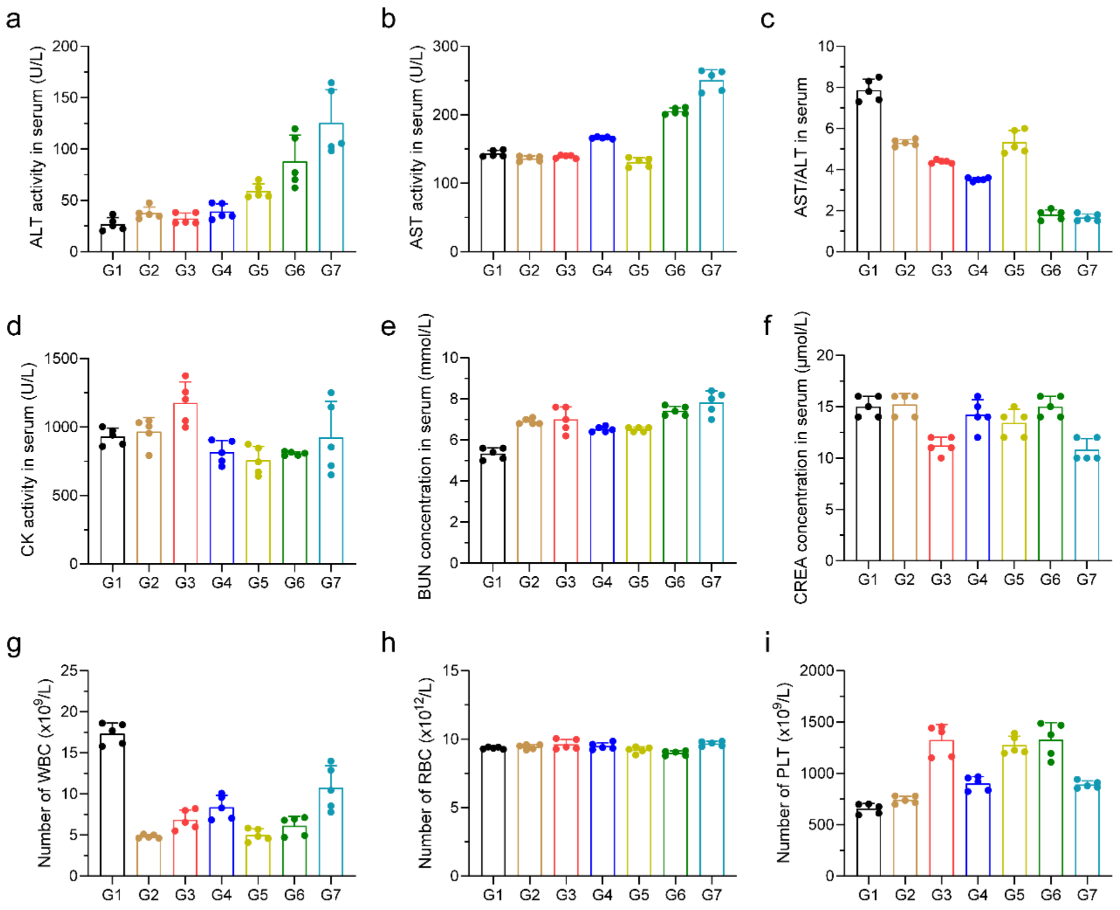

Supplementary Figure 28. Safety of treatment by different combination of RES-blockade and drug delivery strategy. a-f Blood biochemical analysis and **g-i** blood routine examine after treatment. Data are presented as mean values ± SD (*n* = 5 biological independent replicates). G1, Control; G2, Free DOX; G3, DOX@2%NGs; G4, DOX@15%NGs; G5, 2%-blockade + DOX@2%NGs; G6, 15%-blockade + DOX@2%NGs; G7, 15%-blockade + DOX@15%NGs. Source data are provided as a Source Data file.

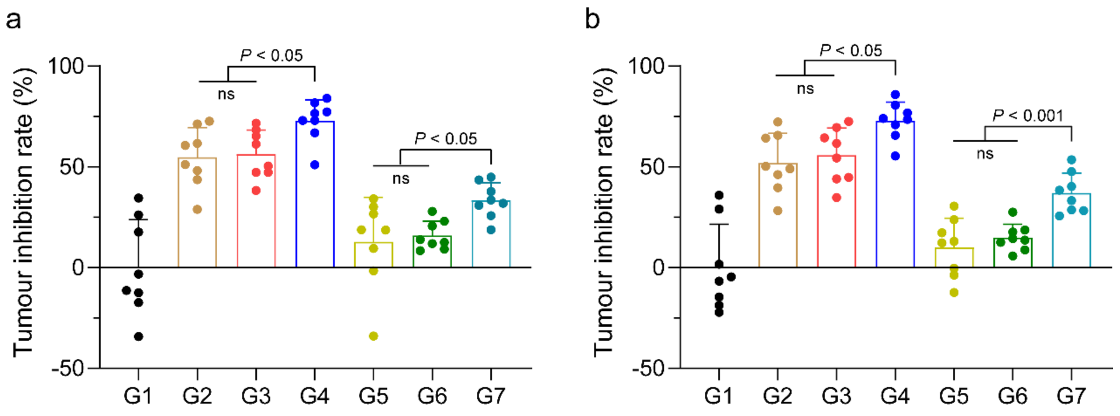

Supplementary Figure 29. Tumour inhibition rate of commercialized nanomedicine

with RES-blockade by soft or stiff nanogels. a Volume-based and **b** weight-based tumour inhibition rate. Data are presented as mean values ± SD (*n* = 8 biological independent replicates). Statistical significance was calculated by unpaired two-sided Student’s t-test. G1, Control; G2, Doxil; G3, 2%-blockade + Doxil; G4, 15%-blockade + Doxil; G5, Abraxane; G6, 2%-blockade + Abraxane; G7, 15%-blockade + Abraxane. Source data are provided as a Source Data file.

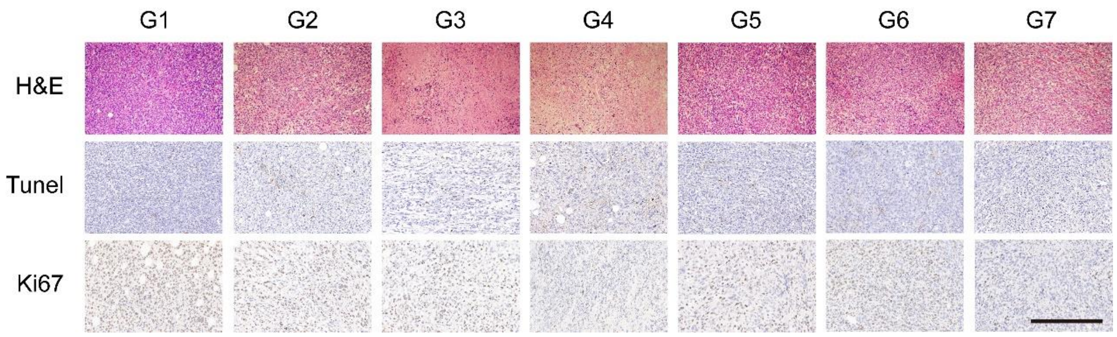

Supplementary Figure 30. Apoptosis and proliferation of tumour cells after treatment by commercialized nanomedicine with RES-blockade by soft or stiff nanogels. H&E, Tunel and Ki67 staining of tumours. Scale bar = 500 μm. G1, Control; G2, Doxil; G3, 2%-blockade + Doxil; G4, 15%-blockade + Doxil; G5, Abraxane; G6, 2%-blockade + Abraxane; G7, 15%-blockade + Abraxane.

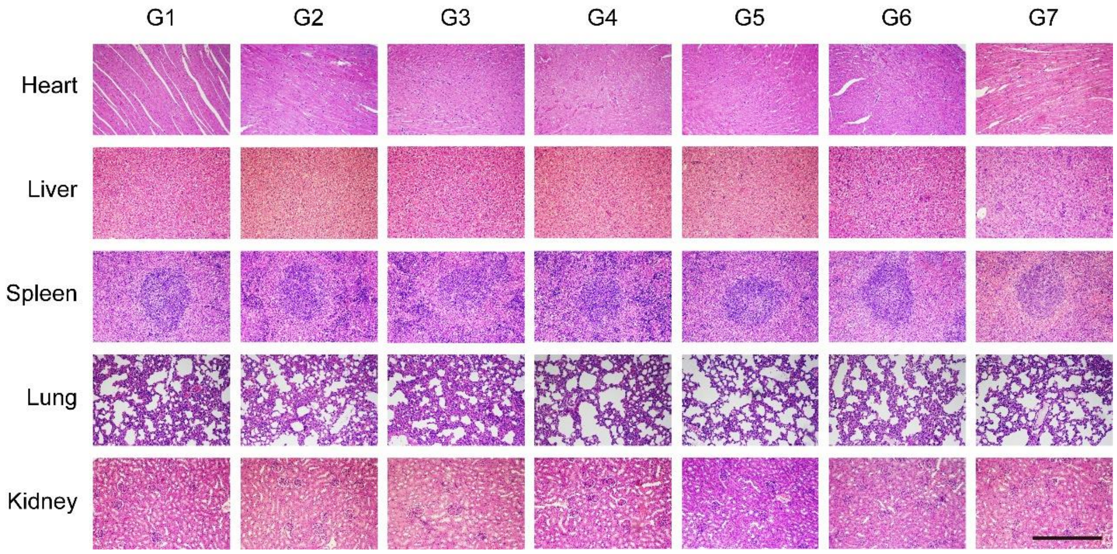

Supplementary Figure 31. Safety of treatment by commercialized nanomedicine with RES-blockade by soft or stiff nanogels. H&E staining of major organs, including heart,

liver, spleen, lung and kidney. Scale bar = 500 μm. G1, Control; G2, Doxil; G3, 2%-blockade + Doxil; G4, 15%-blockade + Doxil; G5, Abraxane; G6, 2%-blockade + Abraxane; G7, 15%-blockade + Abraxane.

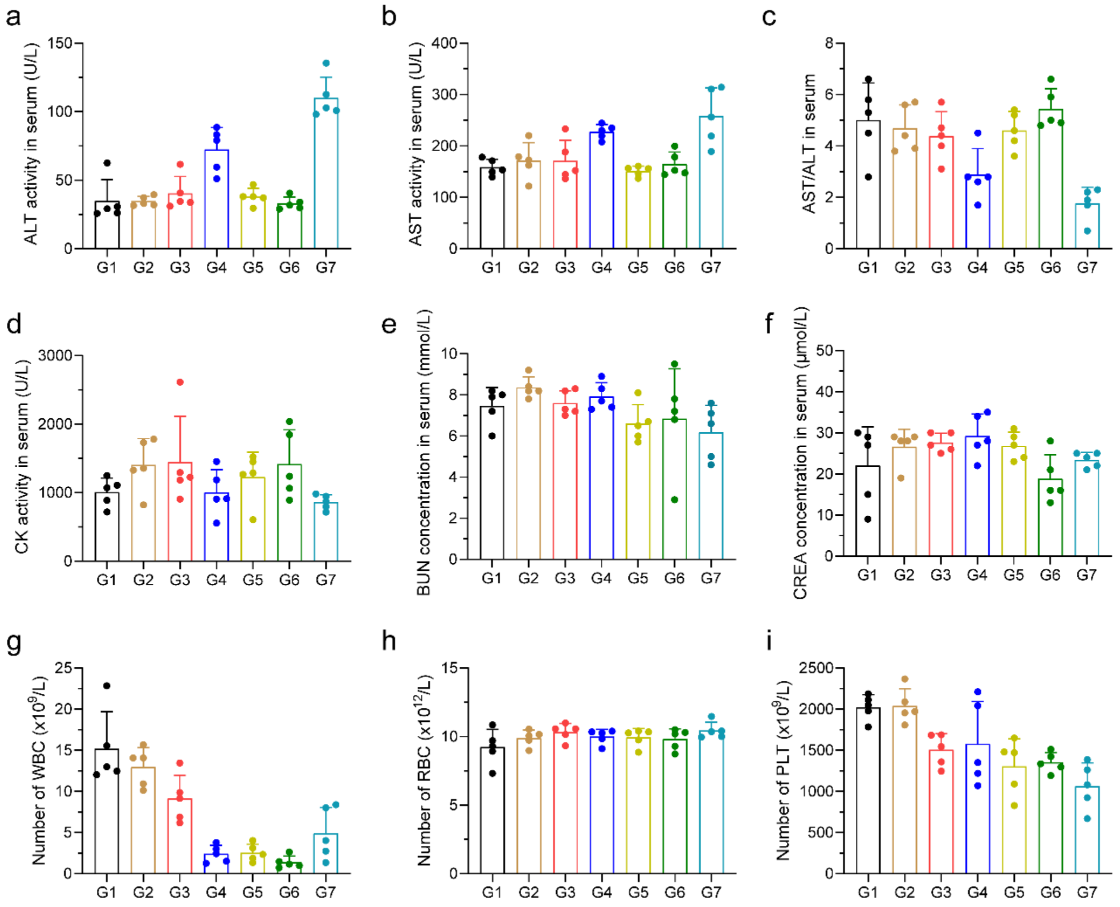

Supplementary Figure 32. Safety of treatment by commercialized nanomedicine with RES-blockade by soft or stiff nanogels. a-f Blood biochemical analysis and **g-i** blood routine examine after treatment. Data are presented as mean values ± SD (*n* = 5 biological independent replicates). G1, Control; G2, Doxil; G3, 2%-blockade + Doxil; G4, 15%-blockade + Doxil; G5, Abraxane; G6, 2%-blockade + Abraxane; G7, 15%-blockade + Abraxane. Source data are provided as a Source Data file.
