# Supplementary material for: Mechano-boosting nanomedicine antitumour efficacy by blocking the reticuloendothelial system with stiff nanogels
Source: Nat Commun. 2023 Mar 15;14:1437. doi: 10.1038/s41467-023-37150-3 (PMC10015032; doi:10.1038/s41467-023-37150-3)
Supplement: Supplementary file 3 — Reporting Summary [file 41467_2023_37150_MOESM3_ESM.pdf]

nature portfolio

Corresponding author(s): Zifu Li

Last updated by author(s): Mar 2, 2023

Reporting Summary

Nature Portfolio wishes to improve the reproducibility of the work that we publish. This form provides structure for consistency and transparency in reporting. For further information on Nature Portfolio policies, see our Editorial Policies and the Editorial Policy Checklist.

Statistics

For all statistical analyses, confirm that the following items are present in the figure legend, table legend, main text, or Methods section.

n/a	Confirmed	
☐	☒	The exact sample size (*n*) for each experimental group/condition, given as a discrete number and unit of measurement
☐	☒	A statement on whether measurements were taken from distinct samples or whether the same sample was measured repeatedly
☐	☒	The statistical test(s) used AND whether they are one- or two-sided<br>*Only common tests should be described solely by name; describe more complex techniques in the Methods section.*
☒	☐	A description of all covariates tested
☒	☐	A description of any assumptions or corrections, such as tests of normality and adjustment for multiple comparisons
☐	☒	A full description of the statistical parameters including central tendency (e.g. means) or other basic estimates (e.g. regression coefficient) AND variation (e.g. standard deviation) or associated estimates of uncertainty (e.g. confidence intervals)
☐	☒	For null hypothesis testing, the test statistic (e.g. *F*, *t*, *r*) with confidence intervals, effect sizes, degrees of freedom and *P* value noted<br>*Give P values as exact values whenever suitable.*
☒	☐	For Bayesian analysis, information on the choice of priors and Markov chain Monte Carlo settings
☒	☐	For hierarchical and complex designs, identification of the appropriate level for tests and full reporting of outcomes
☒	☐	Estimates of effect sizes (e.g. Cohen's *d*, Pearson's *r*), indicating how they were calculated

Our web collection on statistics for biologists contains articles on many of the points above.

Software and code

Policy information about availability of computer code

Data collection	Size and zeta potential were collected with Zetasizer Software (Ver.7.13). AFM images were collected with NanoScope Analysis (Ver.1.5). Flow cytometric data was collected with CytExpert (Ver.2.4). Ex vivo images were collected with Olympus FluoView31S (Ver.2.3). In vivo images were collected with Living Image (Ver.4.0). Nanoparticle tracking analysis data was collected with NanoSight NTA (Ver.3.3).
Data analysis	Size and zeta potential were analyzed with Zetasizer Software (Ver.7.13). Young's modulus was analyzed with NanoScope Analysis (Ver.1.5). Flow cytometric data was analyzed with CytExpert (Ver.2.4). Semi-quantification of in vivo images was analyzed with Living Image (Ver.4.0). Semi-quantification of images was analyzed with ImageJ (Ver.2.0). Statistical analysis was performed using GraphPad Prism 8.Pharmacokinetic was analyzed with Data Analysis Sysem (Ver. 2.0).

For manuscripts utilizing custom algorithms or software that are central to the research but not yet described in published literature, software must be made available to editors and reviewers. We strongly encourage code deposition in a community repository (e.g. GitHub). See the Nature Portfolio guidelines for submitting code & software for further information.

March 2021

Data

Policy information about availability of data

All manuscripts must include a data availability statement. This statement should provide the following information, where applicable:

- Accession codes, unique identifiers, or web links for publicly available datasets
- A description of any restrictions on data availability
- For clinical datasets or third party data, please ensure that the statement adheres to our policy

The authors declare that data supporting the findings of this study are available within the Article, Supplementary Information and Source Data File. Source data are provided with this paper.
Source data are available for Figs. 2a, 2d-f, 3a-c, 3g-I, 3k, 4b, 4d, 4f, 4h, 5b, 5d-g, 5i, 6b-c, 6e-g, 7b-c, 7e-g and Supplementary Figs. 1, 2a-b, 2e-f, 3, 4b, 6b, 7b, 8, 9b-c, 10b, 12, 13, 14b, 15b-c, 15e-f, 16b-c, 17, 18b, 19b-c, 20c-d, 21, 22, 23b-c, 23e-f, 24, 25, 28, 29, 32 in the associated source data file.

Human research participants

Policy information about studies involving human research participants and Sex and Gender in Research.

Reporting on sex and gender	n/a
Population characteristics	n/a
Recruitment	n/a
Ethics oversight	n/a

Note that full information on the approval of the study protocol must also be provided in the manuscript.

Field-specific reporting

Please select the one below that is the best fit for your research. If you are not sure, read the appropriate sections before making your selection.

☒ Life sciences ☐ Behavioural & social sciences ☐ Ecological, evolutionary & environmental sciences

For a reference copy of the document with all sections, see nature.com/documents/nr-reporting-summary-flat.pdf

Life sciences study design

All studies must disclose on these points even when the disclosure is negative.

Sample size	In in vivo and ex vivo imaging experiments, 4-5 mice each group were used to analyze fluorescent intensity of tumour and liver, to ensure repeatability and accuracy of data, and avoid waste of experimental mice.<br>In antitumor experiments, 8 mice each group were used to analyze tumour volume, 8 mice each group were used to analyze tumour weight and tumour inhibition rate, to overcome individual difference among mice and guarantee the statistical significance of data.<br>For other experiments, the sample size for each group was 3, relying on good repeatability of data.
Data exclusions	No data was excluded in the analysis.
Replication	Experiments were independently repeated as indicated and experimental findings were reproducible (described in figure legends for further details).
Randomization	All samples were randomly allocated into experimental groups.
Blinding	Investigators were blinded during tumour inoculation, animal grouping. However, investigators were not blinded to animal drug treatment and data collect of tumour volume and weight. To avoid influence of subjective factors, the investigator was blind to the data collected the day before and another investigator was involved to collect the data once again to evaluate the accuracy of data.<br>As for in vivo imaging experiments, investigators were blinded during tumour inoculation, animal grouping, image capture and data analysis.

Reporting for specific materials, systems and methods

We require information from authors about some types of materials, experimental systems and methods used in many studies. Here, indicate whether each material, system or method listed is relevant to your study. If you are not sure if a list item applies to your research, read the appropriate section before selecting a response.

Materials & experimental systems

n/a	Involved in the study
☐	☒ Antibodies
☐	☒ Eukaryotic cell lines
☒	☐ Palaeontology and archaeology
☐	☒ Animals and other organisms
☒	☐ Clinical data
☒	☐ Dual use research of concern

Methods

n/a	Involved in the study
☒	☐ ChIP-seq
☐	☒ Flow cytometry
☒	☐ MRI-based neuroimaging

Antibodies

Antibodies used	FITC anti-mouse CD31 Antibody (Biolegend, 102405)<br>Recombinant anti-Ki67 Antibody (abcam, ab16667)
Validation	All antibodies were verified by the manufacturers and each lot has been quality tested. All validation statements can be found on the respective antibody website:<br>FITC anti-mouse CD31 https://www.biolegend.com/en-us/products/fitc-anti-mouse-cd31-antibody-120?GroupID=BLG1566<br>Anti-Ki67 https://www.abcam.com/ki67-antibody-sp6-ab16667.html

Eukaryotic cell lines

Policy information about cell lines and Sex and Gender in Research

Cell line source(s)	4T1 cell line was purchased from Shanghai Institutes for Biological Sciences (Shanghai, China). The NIH/3T3 cell line and the murine macrophage cell line RAW264.7 were acquired from National Collection of Authenticated Cell Cultures (Shanghai, China).
Authentication	Each cell line we used was morphologically confirmed according to the information provided by the cell-source center.
Mycoplasma contamination	Cells were tested negative for mycoplasma contamination by using the MycAway-Color one-step mycoplasma detection kit.
Commonly misidentified lines<br>(See ICLAC register)	No commonly misidentified cell lines were used.

Animals and other research organisms

Policy information about studies involving animals; ARRIVE guidelines recommended for reporting animal research, and Sex and Gender in Research

Laboratory animals	BALB/c mice (female, 18 ± 1g, seven-week-old) were purchased from Vital River Laboratory Animal Technology Co. Ltd. (Beijing, China).
Wild animals	No wild animals were used.
Reporting on sex	The findings did not only apply to female mice, female mice were only used for establishment of 4T1 subcutaneous tumour model.
Field-collected samples	No filed-collection was performed.
Ethics oversight	All animal experiments were approved by the Institutional Animal Care and Use Committee at Tongji Medical College, Huazhong University of Science and Technology (Wuhan, China). The experiment protocols were approved by the Institutional Animal Ethical Committee of the Huazhong University of Science and Technology. The animal ethical clearance project number is 2019S924.

Note that full information on the approval of the study protocol must also be provided in the manuscript.

Flow Cytometry

Plots

Confirm that:

☒ The axis labels state the marker and fluorochrome used (e.g. CD4-FITC).

☒ The axis scales are clearly visible. Include numbers along axes only for bottom left plot of group (a 'group' is an analysis of identical markers).

☒ All plots are contour plots with outliers or pseudocolor plots.

☒ A numerical value for number of cells or percentage (with statistics) is provided.

Methodology

Sample preparation	Cultured cells were trypsinized, washed with PBS for three time, and then cells were collected for cytometric analysis.
Instrument	CytoFLEX S.
Software	CytExpert (Ver.2.4).
Cell population abundance	No cell sorting was performed.
Gating strategy	Cells were gated by FSC/SSC gates to select single cells. Then the fluorescent intensity of PE-H was analyzed.

☒ Tick this box to confirm that a figure exemplifying the gating strategy is provided in the Supplementary Information.
